# A Retrospective Observational Study of Insulin Glargine in Type 2 Diabetic Patients with Advanced Chronic Kidney Disease

**DOI:** 10.7759/cureus.6191

**Published:** 2019-11-18

**Authors:** Anirban Majumder, Soumyabrata RoyChaudhuri, Debmalya Sanyal

**Affiliations:** 1 Endocrinology, Kali Prasad Chowdhury Medical College & Hospital, Kolkata, IND

**Keywords:** chronic kidney disease, diabetic kidney disease, efficacy, insulin glargine, safety

## Abstract

Background

The majority of type 2 diabetes mellitus (T2DM) subjects are on multiple oral antidiabetic drugs (OADs) but as kidney dysfunction progresses, many of them become inappropriate. Basal insulin, such as glargine, is generally recommended as first-line insulin therapy by most guidelines. However, there is limited data on the safety and efficacy of the use of glargine in diabetic kidney disease (DKD).

Objectives

To evaluate the efficacy and safety of insulin glargine in T2 DM patients with Stage 3 or 4 chronic kidney disease (CKD).

Material and methods

This single-centered, retrospective, observational study evaluates the efficacy and safety of insulin glargine in DKD with estimated glomerular filtration rate (eGFR) 60 and below. Non-pregnant T2DM patients with DKD receiving insulin glargine for 24 weeks and beyond were included for analysis. Data relating to anthropometric measurements, blood pressure, renal parameters, and glycemic control were analyzed. Sixty patients were in CKD Stage 3 (group A) and 35 patients were in CKD Stage 4 (group B). Glargine was started at an initial dose of 10 units daily as per the standard of care followed by the institute and up-titrated or down-titrated using a prespecified algorithm to maintain fasting plasma glucose between 90 mg/dl and 130 mg/dl.

Results

The study achieves (1.2%) (13.2 mmol/mol) of glycosylated hemoglobin (HbA1C) reduction in both groups (Group A and Group B) and a significant reduction in fasting and postprandial glucose values without a significant weight change over the study period. Out of 95 patients, 32 (33.68%) had documented hypoglycemia; out of them, 9 (28.2%) had severe hypoglycemia, and 8 (25%) had nocturnal hypoglycemia (either mild or severe). No change in weight, blood pressure, or eGFR was observed during the study period.

Conclusions

Treatment with glargine-based basal insulin therapy in diabetes with Stage 3 or Stage 4 CKD was efficacious in reducing glycemic parameters and was safe without significant changes in weight and hypoglycemia.

## Introduction

The prevalence of type 2 diabetes mellitus (T2DM) is ever-increasing, with projections that one out of every five diabetics will be an Indian by the year 2025 [1]. The magnitude of multi-organ damage in T2DM is highly acknowledged, amongst which renal involvement is extremely critical. Diabetic kidney disease (DKD), if not properly managed, can progress to more advanced stages of chronic kidney disease (CKD) [2]. Further, patients with CKD, particularly with T2DM, are the ones at a higher risk of cardiovascular events. Once CKD is established, clinicians should determine the optimal glycemic targets [3] and treat accordingly, to reduce microvascular and macrovascular complications [4]. Reaching optimal glycemic targets are often complicated due to increasing hypoglycemic episodes, as DKD subjects are prone to hypoglycemia [5]. There is a paucity of data regarding ideal insulin preparations in DKD subjects owing to the lack of pharmacokinetic-pharmacodynamics studies for various insulin preparations in subjects with varying magnitudes of renal insufficiency [6-7].

Long-acting insulin analogs have comparatively flat pharmacokinetic profiles and a longer duration of action [8]. The efficacy and safety of insulin glargine, a long-acting insulin analog, is well-established in both type 1 diabetes mellitus (T1DM) and T2DM [9]. It provides an effective basal insulin supply [10-11] and has a lower propensity to cause hypoglycemia as compared to neutral protamine Hagedorn (NPH) insulin, with similar efficacy in glycemic control. There is no consensus on the usage of glargine in subjects with advanced renal failure, possibly due to a paucity of published data [7,12]. However, insulin glargine is commonly used in clinical practice among subjects of T2DM with DKD, despite uncertainty about the efficacy and safety profile in these subjects. The present retrospective study is aimed to assess the efficacy and safety of insulin glargine in T2 DM subjects with established CKD.

## Materials and methods

Study design

This was a single-centered, retrospective, real-world observational study to evaluate the efficacy and safety of insulin glargine in T2DM with CKD. The data of 95 patients who were taking insulin glargine for a period of at least 24 weeks in the endocrine outpatient department (OPD) of KPC Medical College & Hospital, Kolkata, have been presented in the study. The study period was from July 2016 to June 2018.

Inclusion and exclusion criteria

The included study patients were non-pregnant, T2DM, with estimated glomerular filtration rate (eGFR) between 15 and 59 ml (CKD Stages 3 and 4) who failed to achieve target glycemic control with oral antidiabetic drug or prandial insulin and received insulin glargine for a period of at least 24 weeks at the time of recording their data. We excluded all patients with T1DM or diabetic ketoacidosis, alcohol or drug dependence, recent or multiple hospitalization for reasons other than hyperglycemia within the past six months, nursing women, urinary tract or other systemic infections, hematuria, decompensated heart failure, liver failure, debilitating illness that may adversely affect renal function, or on drugs that may adversely affect renal function.

Participants were evaluated for CKD on the basis of eGFR. Patients with CKD Stages 3 and 4 were included in the present study (Table 1 ). Patients who were not optimally controlled on oral antidiabetic drugs (OADs), with or without short-acting regular human insulin, received insulin glargine as the standard of care in our OPD. Patients not optimally controlled were defined as either having one or more of the following: fasting plasma glucose (FPG) > 150 mg/dL, post-prandial plasma glucose (PPPG) > 200 mg/dL, HbA1c > 8.5% (69.5 mmol/mol) despite receiving optimal dose of two or three OADs and with or without prandial insulin [13]. The starting dose of glargine was 10 units daily, and it was monitored and titrated at prespecified intervals according to the value of the self-monitoring of blood glucose (SMBG) to obtain target fasting capillary blood glucose (CBG less than 130mg/dl). All patients received treatment as per the routine standard of care. Based on existing guidelines, the main components of our hospital out-patient protocol are: adoption of basal insulin administration starting with 10 units daily dose; regular monitoring of blood glucose (at least weekly); algorithm for basal insulin dose adjustment based on fasting capillary blood glucose; achievement of a pre-meal glucose target of less than 130 mg/dl and an average day-time of less than 180 mg/dl [14]. However, a lower target range is usually set for patients who are able to achieve and maintain better glycemic control without hypoglycemia.

**Table 1:** Staging of CKD Adapted from: American Diabetes Association. Standards of medical care in diabetes. Diabetes Care. 2014; 37; S14-S80 GFR: glomerular filtration rate; CKD: chronic kidney disease

CKD Stage	Description	GFR (mL/min/1.73 m^2^ body surface area)
1	Kidney damage* with normal or increased GFR	>=90
2	Kidney damage* with mildly decreased GFR	60–89
3	Moderately decreased GFR	30–59
4	Severely decreased GFR	15–29
5	Kidney failure	<15 or dialysis
*Kidney damage defined as abnormalities on pathological, urine, blood, or imaging tests.		

Treatment algorithm for diabetes with CKD

Step 1: Determine the individualized glycemic target.

Target range: Target range: (Based on patient preference, cardiovascular comorbidities, and hypoglycemia risk) in most situations, fasting capillary blood glucose < 130 mg/dl and HbA1c: 7.5%-8 % (58.5-63.9 mmol/mol).

Step 2: Initiate glargine in a 10-unit night dose if the glycemic control is not on target. All oral antidiabetic drugs to be continued if not contraindicated as per eGFR. Only renal-safe sulfonylurea (SU; Glipizide/Gliclazide) to be continued and all other SUs to be stopped. 

Patient instruction: Monitor CBG in a given log sheet, fasting and pre-dinner weekly and randomly, if symptoms of hypoglycemia exist. Contact hospital emergency in severe and unexplained hypoglycemia.

Step 3: If fasting capillary blood glucose is above the patient's target range, the dosage of glargine should be increased by two units weekly unless there is no severe and unexplainable hypoglycemia. If fasting capillary blood glucose is below the patient's target range, decrease the dosage of glargine by two units weekly.

Data collection

Data of all patients fulfilling the inclusion and exclusion criteria and managed according to our protocol in the OPD were retrospectively collected from our hospital OPD records. Data included all patients’ demographic records with respect to age, gender, body weight, blood pressure (BP), and duration of diabetes. Data were collected on the day of starting insulin glargine and at least 24 weeks after starting glargine. All laboratory investigations data for HbA1c, FPG, PPG, serum creatinine (Cr), eGFR, urine albumin-to-creatinine ratio (UACR), sodium (Na), and potassium (K) were also recorded on the day of starting insulin glargine and at least 24 weeks after starting glargine. Plasma glucose was measured by the hexokinase method and HbA1c was measured by the high-performance liquid chromatographic (HPLC) method (Bio-RAD D-10, Bio-RAD Inc., CA, USA) in our hospital. Only those patients were considered for the final evaluation who had both baseline and post-treatment values of the study parameters.

Renal function was determined by eGFR using the modification of diet in renal disease study (MDRD) equation. The stages of nephropathy were classified according to the international standard, as recommended by the American Diabetes Association (Table 1).

Due to the observational study design used to assess the efficacy and safety of insulin glargine in T2DM with established DKD, there was no randomization of the subjects.

Visit and assessments

Baseline and 24 weeks or beyond follow-up.

Efficacy assessment

Laboratory parameters.

Safety assessment

Information about all adverse events, such as hypoglycemia (documented, severe, and nocturnal), allergic rashes, or local injection site reaction that might have occurred during the period were recorded by interviewing the patients on the last follow-up visit. As the standard care of the outpatients of the hospital, all diabetics on insulin are provided with a capillary blood glucose log chart to record fasting and pre-dinner SMBG weekly and when symptoms of hypoglycemia occur. Data from the log were captured as a hypoglycemia episode when the value is below the set definition of hypoglycemia.

Hypoglycemia was defined as a capillary glucose level of ≤ 70 mg/dl. An episode during which typical symptoms of hypoglycemia were accompanied by a measured capillary glucose concentration ≤ 70 mg/dl is considered documented hypoglycemia. Patients with transient dysfunction of the central nervous system who were unable to treat themselves (requiring third-party assistance) were considered to have severe hypoglycemia [15-16]. Nocturnal hypoglycemia was defined as an episode of abnormally low capillary glucose levels (< 54 mg/dL) occurring at night (12 AM to 6 AM) during sleep as per the International Hypoglycemia Study Group [16].

Ethical issue

All patients received treatment as per the routine standard of care. Patients receiving and continuing glargine for more than six months were invited to participate in the study and their data were recorded for analysis. Written informed consent was obtained from all patients prior to recording their data. The uploaded data in the electronic database for analysis did not contain the patient’s contact number, address, or any other confidential information that was not pertaining to the study objectives. The study was approved by the ethics committee of the institute.

Statistical methods

A descriptive statistical analysis was carried out in the present study. Results on continuous measurements were presented as mean ± standard error of the mean (SEM) and results on categorical measurements were presented in number (%). Significance was assessed at a level of 5%. The normality of data was tested by a simultaneous Anderson Darling test, Shapiro-Wilk test, and graphically by the QQ plot. The paired t-test was implemented to find any significant changes in the study parameters from baseline to follow-up for the same group of patients.

Statistical software

Statistical software, namely SAS (Statistical Analysis System) version 9.2 for Windows (SAS Institute Inc., NC, US) and Statistical Package for Social Sciences (SPSS Complex Samples) version 21.0 for Windows (SPSS, Inc., IL, US) were used for the analysis of the data. Microsoft Word 2010 and Microsoft Excel 2010 (Microsoft Corp, Redmond, WA, US) were used to generate graphs and tables.

## Results

All the 95 patients included in the study were divided into two groups on the basis of eGFR (Group A: CKD Stage 3 and Group B: CKD Stage 4). Sixty patients (Group A) included in the study showed a moderate (30-59 ml/min) and 35 (Group B) a severe reduction of eGFR (15-29 ml/min).

A summary of the demographic and laboratory data of the total 95 patients and the two sub-groups is given in Table 2. There is no relevant difference between the subgroups concerning age, sex, duration of diabetes, diastolic BP, fasting capillary glucose, post-prandial capillary glucose, HbA1c, UACR, sodium, and potassium. There was a significant difference between the two groups in body weight, systolic BP, and serum creatinine and eGFR levels.

**Table 2:** Baseline characteristics of 95 patients with renal dysfunction P<0.05 considered as statistically significant, p computed by unpaired t-test CKD: chronic kidney disease; SEM: standard error of the mean; SBP: systolic blood pressure; DBP: diastolic blood pressure; HbA1c: glycosylated hemoglobin; ACR: albumin to creatinine ratio; eGFR: estimated glomerular filtration rate

	Total Cohort, N=95	Group A CKD Stage 3, N=60	Group B CKD Stage 4, N=35	p
Male, n	60	42	18	0.054
Female, n	35	18	17	
Age (years), Mean ± SEM	62.1 ± 1.34	61.6 ± 1.47	63.0 ± 1.59	0.535
Duration of diabetes (years), Mean ± SEM	14.7 ± 0.96	13.2 ± 1.02	15.4 ± 1.48	0.166
Bodyweight (Kg), Mean ± SEM	66.7 ± 1.16	71.1 ± 1.46	59.2 ± 1.04	<0.001
SBP (mm Hg), Mean ± SEM	150.9 ± 2.73	146.0 ± 2.55	163 ± 5.61	0.002
DBP (mm Hg), Mean ± SEM	81.3 ± 1.03	81.2 ± 1.15	80.7 ± 2.56	0.83
Fasting capillary glucose (mg/dl), Mean ± SEM	181.2 ± 7.54	182.9 ± 10.52	178.2 ± 9.94	0.762
Post-prandial capillary glucose (mg/dl), Mean ± SEM	262.9 ± 10.41	249.4 ± 11.54	285.1± 15.09	0.065
HbA1c (%), Mean ± SEM	8.7 ± 0.24	8.8 ± 0.28	8.7 ± 0.29	0.086
Urine ACR, Mean ± SEM	702.3± 106.94	587.4 ± 106.68	922.8± 231.98	0.058
Serum creatinine (mg/dl), Mean ± SEM	2.0 ± 0.08	1.7 ± 0.04	2.6 ± 0.12	<0.001
eGFR (mL/min/1.73 m^2^), Mean ± SEM	34.9 ± 1.21	41.8 ± 1.09	23.3 ± 0.97	<0.001
Serum sodium, mEq/L	133.9 ± 0.83	134.7 ± 0.86	131.2 ± 9.81	0.544
Serum potassium, mEq/L	4.0 ± 1.74	3.4 ± 0.84	4.7 ± 2.51	0.145

As shown in Tables 3-4, there were no statistically significant changes concerning the body weight, systolic BP, and diastolic BP in the total population and in the subgroup over the 24±4.2 weeks follow-up.

**Table 3:** Changes in anthropometric study parameters values in 95 patients with renal dysfunction (CKD Stage 3 and CKD Stage 4) P<0.05 considered as statistically significant, p computed by paired t-test CKD: chronic kidney disease; SEM: standard error of the mean; SBP: systolic blood pressure; DBP: diastolic blood pressure

Anthropometric Parameters	Baseline	Follow-up (24±4.2 weeks)	p-value (two-tailed)
	Mean ± SEM	Mean ± SEM	
Body Weight, kg	66.7 ± 1.16	66.6 ± 1.07	0.612
SBP, mmHg	150.9 ± 2.73	148.7 ± 2.32	0.269
DBP, mmHg	81.3 ± 1.03	81.0 ± 0.93	0.943

**Table 4:** Changes in anthropometric study parameters: sub-group analysis by CKD stage P<0.05 considered as statistically significant, p computed by paired t-test CKD: chronic kidney disease

CKD Staging	Anthropometric Parameters	Baseline	Follow-up (24 ± 4.2 weeks)	p-value (two-tailed)
		Mean ± SEM	Mean ± SEM	
CKD Stage 3, N=60	Body Weight, kg	71.1 ± 1.46	70.6 ± 1.37	0.242
	SBP, mmHg	146.0 ± 2.55	144.5 ± 1.89	0.481
	DBP, mmHg	81.2 ± 1.15	81.5 ± 0.93	0.405
CKD Stage 4, N=35	Body Weight, kg	59.2 ± 1.04	59.6 ± 1.07	0.303
	SBP, mmHg	163 ± 5.61	158.8 ± 1.05	0.397
	DBP, mmHg	80.7 ± 2.56	78.3 ± 2.15	0.304

Out of 95 patients included in the study, nearly half of the participants (49%) were taking linagliptin, 35% were taking short-acting insulin, 18% used metformin, 16% used repaglinide, 12% used glimepiride, 8% used glipizide, 10% used gliclazide, and 6% used alpha-glucosidase inhibitors when glargine was started. When glargine was started, all oral hypoglycaemic agents (OHAs) were continued but short-acting insulins were withdrawn.

Overall, the mean fasting capillary glucose levels changed from 181.2 mg/dl to 115.6 mg/dl (p 0.018), mean post-prandial capillary glucose levels changed from 262.9 mg/dl to 191.5 mg/dl (p 0.047), and mean HbA1c levels changed from 8.7% (72 mmol/mol) to 7.5% (59 mmol/mol) (p < 0.001) over the 24±4.2 weeks follow-up (Table 5).

**Table 5:** Changes in glycemic study parameters values in 95 patients with renal dysfunction p<0.05 considered as statistically significant; HbA1c: glycosylated hemoglobin

Glycemic Parameters	Baseline	Follow-up (24±4.2 weeks)	p-value (two-tailed)
	Mean ± SEM	Mean ± SEM	
Fasting capillary glucose, mg/dL	181.2 ± 7.54	115.6 ± 3.26	0.018
Post prandial capillary glucose, mg/dL	262.9 ± 10.41	191.5 ± 4.63	0.047
HbA1c, %	8.7± 0.24	7.5 ± 0.13	<0.001

The mean change in fasting capillary glucose, post-prandial capillary glucose and HbA1c levels among the subgroups (group A and group B) are shown in Table 6.

**Table 6:** Changes in glycemic study parameters values: sub-group analysis by CKD staging CKD: chronic kidney disease; SEM: standard error of the mean; HbA1c: glycosylated hemoglobin

CKD stage	Glycemic parameters	Baseline	Follow-up (24 ± 4.2 weeks)	p-value (two-tailed)
		Mean ± SEM	Mean ± SEM	
Stage 3	Fasting capillary glucose, mg/dL	182.9 ± 10.52	121.1 ± 4.47	<0.001
	Post-prandial capillary glucose, mg/dL	249.4 ± 11.54	187.7 ± 5.96	<0.001
	HbA1c, %	8.8 ± 0.28	7.6 ± 0.15	<0.001
Stage 4	Fasting capillary glucose, mg/dL	178.2 ± 9.94	106.4 ± 4.07	<0.001
	Post-prandial capillary glucose, mg/dL	285.1 ± 15.09	197.6 ± 7.36	<0.001
	HbA1c, %	8.7 ± 0.29	7.4 ± 0.22	<0.001

Changes in renal function parameters were also evaluated over the study period. Overall, the mean urine ACR levels decreased from 702.3 mg/gm to 446.6 mg/gm (p 0.001) and mean serum sodium levels increased from 133.9 mEq/L to 138.0 mEq/L (p 0.032) significantly. The other renal parameters (serum creatinine, eGFR, and potassium) had not changed significantly over the 24±4.2 weeks follow-up after starting glargine therapy (Table 7).

**Table 7:** Changes in renal function study parameters values in 95 patients with renal dysfunction p<0.05 considered as statistically significant ACR: albumin to creatinine ratio; eGFR: estimated glomerular filtration rate

Renal function tests	Baseline	Follow-up (24±4.2 weeks)	p-value (two-tailed)
	Mean ± SEM	Mean ± SEM	
Urine ACR, mg/g	702.3 ± 106.94	446.6 ± 104.51	0.001
Serum creatinine, mg/dL	2.0 ± 0.08	1.9 ± 0.05	0.885
eGFR (mL/min/1.73 m2)	34.9 ± 1.21	36.0 ± 1.36	0.057
Serum sodium, mEq/L	133.9 ± 0.83	138.0 ± 0.73	0.032
Serum potassium, mEq/L	4.0 ± 1.74	3.1 ± 0.05	0.500

The mean change in renal parameters among the subgroups (group A and group B) is shown in Table 8.

**Table 8:** Changes in renal function study parameters values: sub-group analysis by CKD staging CKD: chronic kidney disease; SEM: standard error of the mean; ACR: albumin to creatinine ratio; eGFR: estimated glomerular filtration rate

CKD stage	Renal function tests	Baseline	Follow-up (24 ± 4.2 weeks)	p-value (two-tailed)
		Mean ± SEM	Mean ± SEM	
Stage 3	Urine ACR, mg/g	587.4 ± 106.68	292.1 ± 66.97	0.009
	Serum creatinine, mg/dL	1.7 ± 0.04	1.7 ± 0.07	0.057
	eGFR (mL/min/1.73 m2)	41.8 ± 1.09	43.2 ± 1.33	0.188
	Serum sodium, mEq/L	134.7 ± 0.86	137.6 ± 0.89	0.058
	Serum potassium, mEq/L	3.4 ± 0.84	2.9 ± 0.08	0.743
Stage 4	Urine ACR, mg/g	922.8 ± 231.98	762.9 ± 178.46	0.034
	Serum creatinine, mg/dL	2.6 ± 0.12	2.3 ± 0.13	0.326
	eGFR (mL/min/1.73 m2)	23.3 ± 0.97	24.3 ± 1.33	0.039
	Serum sodium, mEq/L	131.2 ± 9.81	129.1 ± 1.22	0.109
	Serum potassium, mEq/L	4.7 ± 2.51	4.1 ± 1.36	0.012

Overall, 33.68% of the patients (32 out of 95) had documented hypoglycemia, out of them, 28.2% had severe and 71.8% had mild hypoglycemia. Nocturnal hypoglycemia (either mild or severe) was documented among 25% of patients (Table 9).

**Table 9:** Incidence of hypoglycemia in the overall cohort

Hypoglycemic episodes/ events	Number of patients	Number of events	Percentage
Total documented hypoglycemia	32	32	100 %
Documented mild hypoglycemia	23	23	71.8 %
Documented severe hypoglycemia	9	9	28.2 %
Nocturnal hypoglycemia ( mild + severe)	8	8	25 %

The hypoglycemic episodes among the subgroups (group A and group B) are analyzed in Table 10, and there is no statistical difference between the two groups.

**Table 10:** Incidence of hypoglycemia by CKD staging

Hypoglycemia episodes/events	CKD stage 3, N=60 number	CKD stage 4, N=35 number	P
Hypoglycemia	9	14	0.201
Severe hypoglycemia	3	6	0.343
Nocturnal hypoglycemia	2	6	0.236

## Discussion

There is conflicting data on the role of glycemic control on the progression of established nephropathy [17]. However, patients with worse control fare poorly and are likely to develop or have deterioration rapidly from their exiting associated complications such as retinopathy and neuropathy. Many international guidelines recommend individualized HbA1c targets. Currently, there is a lack of evidence to guide the use of glucose-lowering agents in people with DKD. In a recently published Cochrane Systematic Review on insulin and glucose-lowering agents for treating people with diabetes and chronic kidney disease, no conclusions could be made for the types, dosages, or modes of administration of insulin. There is also little evidence to evaluate the safety and efficacy of insulin and to guide the choice, type, dosing, and optimal route of administration of insulin. Four studies compared different types, dosages, or modes of administration of insulin. One study [18] compared the efficacy of once-daily glargine plus three-times glulisine at 0.5 vs. 0.25 units/kg/day in those with an eGFR ≤ 45 mL/min/1.73 m2 and showed 0.25 units/kg/day dosing reduced the frequency of hypoglycemia without compromising the control. Another crossover study [19] compared insulin lispro to regular insulin in those with a GFR of 50 to 60 mL/min. One parallel study [20] and one crossover study compared intraperitoneal to subcutaneous insulin in those receiving peritoneal dialysis. None of the studies could be included in the meta-analysis due to heterogeneity in the intervention or presentation of the results.

The present study was a retrospective observational study in patients who were on insulin glargine at least for 24 weeks with Stage 3 and Stage 4 CKD with uncontrolled diabetes. Data on baseline parameters and changes from baseline after 24±4.2 weeks were analyzed. In the present study, HbA1c was significantly reduced from the baseline to the last follow-up visit. The reduction of HbA1c was regardless of the avoidance of tight glycemic control. There is little improvement in overall eGFR (from 34.9±1.21 to 36.0±1.36) but not statistically significant ( Table 7 ). It is difficult to explain the observation. However, a change in lifestyle advice with deteriorating renal function as a standard of care, improvement in overall blood pressure (SBP from 150.9±2.73 to 148.7±2.32 and DBP from 81.3±1.03 to 81.0±0.93) (Table 3), improved glycaemic control (HbA1c from 8.7±0.24 to 7.5±0.13) (Table 5) and other confounding variables, including medications, may have contributed to this benefit. Lately, long-acting insulin analogs have been assessed in uremic patients on hemodialysis. Pscherer et al. described the results of a retrospective clinical study performed on 20 diabetic patients (four type 1 and 16 type 2) with CKD on hemodialysis (time on dialysis approximately 43 months) treated with insulin glargine. Glycaemic control and the incidence of hypoglycemia were analyzed. Nineteen patients had previously been treated with human insulin (conventional or intensive insulin therapy) and one patient with oral agents. All patients were changed to insulin glargine and those patients on conservative insulin therapy were treated with intensive insulin therapy. Insulin glargine doses were individualized and therapy duration was approximately nine months. With this therapeutic regimen, HbA1c was reduced by 0.9% (p<0.01), severe hypoglycaemic events were not reported, and dry weight increased approximately by 1.5 kg [21]. In our study, a significant reduction in HbA1c of 1.2% in 24±4.2 weeks (p<0.001) was observed. The overall reduction of 1.2% in this study was much higher when compared to the Pscherer et al. study [21]. Similar results of reduction in HbA1c were seen in a sub-group analysis of Stage 3 and Stage 4 patients also [22]. A study by Niafar et al. (2012) found a significant HbA1c reduction of 0.71 % (from 8.4% ± 1.6 to 7.7% ± 1.2, p<0.001) after four months of therapy, whereas we observed a higher HbA1c reduction of 1.2% [23].

As GFR declines, clearance by the kidneys is reduced, so doses of insulin may need adjustment. There is also a greater risk of hypoglycemia. In our study, 33.68% of the patients (32 out of 95) had documented hypoglycemia; out of them, 28.2% had severe hypoglycemia, and nocturnal hypoglycemia (either mild or severe) was documented among 25% subjects. The rate of hypoglycemia reported in this study is in line with or similar to that of Pscherer et al., Niafar et al., and the landmark Diabetes Control and Complications Trial (DCCT) [21,23-24].In a recently published prospective study of 89 T2DM patients with eGFR ~ 38±14 ml/min per 1.73 m2 and duration of diabetes ~20±11 years, Ahmad et al. documented 255 episodes of level 1 hypoglycemia (<70 mg/dL), of which 68 episodes reached level 2 hypoglycemia(<54 mg/dL) over a period of 890 days. In the present study, we reported 23 episodes of mild hypoglycemia (comparable to level 1 hypoglycemia) and nine episodes of severe hypoglycemia (comparable to level 2 hypoglycemia) over a period of 24 weeks. Ahmed et al. reported the median rate of hypoglycemia as 5.3 episodes/30 days and the average time spent in hypoglycemia was noted as 28±37 minutes/day. However, one has to account for the difference in the methodology for monitoring of blood glucose between our study (SMPG) and Ahmad et al. by continuous glucose monitoring (CGM). It is well-known that CGM markedly increases the detection of hypoglycemia as compared to SMPG and may not be always feasible in routine clinical practice [25].

Glargine is clinically equivalent to NPH in terms of glycemic control but has advantages in terms of hypoglycemia, especially nocturnal hypoglycemia [26]. It has also been observed that in clinical trials, a single daily injection of insulin glargine provides glycemic control equivalent to NPH insulin [27]. However, people treated with insulin glargine showed a significant improvement in baseline to endpoint in HbA1c as compared to those people treated with NPH insulin at week 20 but with a lower risk of hypoglycemia [28]. Peterson et al. also mentioned that glargine provides better glycemic control than NPH insulin without increasing the risk of hypoglycemia [29].

Baldwin et al. conducted a multicenter, prospective, randomized trial to compare the efficacy and hypoglycemic events of once-daily glargine plus three-times daily glulisine at 0.5 units/kg/day (high dose insulin group ) vs. 0.25 units/kg/day (low dose insulin group) in 107 T2DM subjects with glomerular filtration rate of <45 mL/min but who did not require dialysis [18]. There was equivalent glycemic control between groups (high-dose group vs low-dose group). However, 30% in the high-dose group experienced hypoglycemia (BG<70 mg/dL) as compared with 15.8% (P = 0.08) of the low-dose group. Baldwin et al. concluded that a reduction of initial glargine/glulisine insulin weight-based dosing in hospitalized patients with diabetes and renal insufficiency reduced the frequency of hypoglycemia by 50% without compromising the control of hyperglycemia [18].

Cardiovascular disease in T2DM involves complex pathophysiology that is promoted mostly by traditional risk factors such as obesity, hypertension, dyslipidemia, tobacco smoking, physical inactivity, and so on. Weight gain is an undesirable effect of insulin therapy. Though insulin glargine has been associated with weight gain in most of the studies, many other studies have reported no significant weight gain [19]. There is no statistically significant change in body weight (Table 3) observed over the period of 24±4.2 weeks in our study, rather there is a statistically non-significant minor (0.46 kg) reduction in weight observed. Weight reduction, in this study, showed better results than that reported by Pscherer et al. [21], probably because of advanced CKD.

In the outcome reduction with initial glargine intervention (ORIGIN) trial, no blood pressure differences were seen among T2DM subjects randomized to glargine or standard glycemic care [30]. Similarly, there is no statistically significant change in systolic BP and diastolic BP (Table 3) observed over the period of 24±4.2 weeks in our study also. Combined with the convenience of once-daily injection, the absence of weight gain and a neutral effect on blood pressure, this agent may be a step closer to achieving target glycemic control in patients with T2DM with established CKD. The influence of insulin treatment on the development of diabetes-related microvascular complications has been sparsely investigated. Few studies on T1DM patients with impaired renal function demonstrated better kidney function and a lower urinary albumin/creatinine ratio when treated with insulin analogs, especially Glargine and Lispro. Similarly, a statistically significant reduction in ACR, along with an improvement in glycemic profile, was observed in our study (Table 7). However, no statistically significant change in other renal parameters (serum creatinine, eGFR, and potassium), was observed over the period of 24±4.2 weeks. We observed a significant 4.14 mEq/L (138.07-133.93) rise in Na value on follow-up visit but it was not associated with any significant change in diastolic or systolic blood pressure. The benefit of improving ACR was also observed in the subgroup analysis. This benefit is possibly due to the improvement in overall blood pressure (SBP from 150.9±2.73 to 148.7±2.32 and DBP from 81.3±1.03 to 81.0±0.93) (Table 3), improved glycemic control (HbA1c from 8.7±0.24 to 7.5±0.13) (Table 5) or the result of concomitant medication used (dipeptidyl peptidase-4 inhibitors, for example). This improvement in renal status implies the additional benefits of lowering renal and cardiovascular prognosis in such types of patients. If confirmed in future prospective studies, this may present an opportunity for delaying the progression of diabetic nephropathy.

Limitations of the study

Though almost all the results of this study are in line with published results, retrospective study design, relatively small number of participants, random sample size, non-powered sample size calculation, and lack of comparator arm are significant limitations of this study. Having a small sample size, a propensity score using variables was not done, which might affect the treatment outcomes and is another significant lacuna.

## Conclusions

The treatment of T2DM subjects, with established CKD, with glargine, resulted in a significant reduction in HbA1c, no increase in body weight, and an acceptable risk of hypoglycemic events. Thus, in the background of the paucity of available data on using basal insulin in advanced CKD, the study shows that glargine may be considered a safe and effective option.
